# Supplementary material for: Development of Plasma Protein Classification Models for Alzheimer’s Disease Using Multiple Machine Learning Approaches
Source: Int J Mol Sci. 2025 Dec 2;26(23):11673. doi: 10.3390/ijms262311673 (PMC12692058; doi:10.3390/ijms262311673)
Supplement: Supplementary file 1 [file ijms-26-11673-s001.zip › ijms-3913508-supplementary.pdf]

International Journal of
Molecular Sciences

MDPI

Supplementary Materials

	EBlasso			EBEN		
	**Estimate**	**95% CI**	**p-value**	**Estimate**	**95% CI**	**p-value**
Intercept	0.507	[-1.109, 2.122]	0.539	1.955	[-0.422, 4.333]	0.107
**ANG-2/ANGPT-2**	2.051	[0.101, 4.001]	0.039	2.124	[-0.538, 4.787]	0.118
**EGF**	-0.253	[-1.827, 1.320]	0.752	-0.638	[-2.165, 0.889]	0.413
**IL-1α**	-3.244	[-5.857, -0.631]	0.015	-4.766	[-7.813, -1.719]	0.002
**IL-3**	-1.408	[-3.576, 0.759]	0.203	-1.508	[-4.542, 1.526]	0.330
**IL-11**	3.348	[0.349, 6.346]	0.029	5.516	[1.213, 9.819]	0.012
**PDGF-BB**	-0.836	[-1.990, 0.318]	0.156	-1.027	[-2.338, 0.285]	0.125
**TNF-α**	-2.333	[-4.267, -0.399]	0.018	-1.505	[-4.118, 1.108]	0.259
**BLC**	-----	-----	-----	5.078	[0.665, 9.491]	0.024
**M-CSF/CSF1**	-----	-----	-----	-2.813	[-5.968, 0.342]	0.081

Supplementary Table S1. The coefficient estimates [95% CI] and p-value for the models developed using EBlasso (7 proteins) and EBEN (9 proteins) to select predictors.

International Journal of
Molecular Sciences

MDPI

	Mean SHAP score	
	**XGBoost**	**LightGBM**
**IL-1α**	0.929	0.781
**IL-11**	0.440	0.329
**GCSF**	0.312	0.486
**PDGF-BB**	0.309	0.194
**IL-1RA/IL1RN**	0.265	0.418
**ANG-2/ANGPT-2**	0.243	0.123
**IL-3**	0.213	0.194
**MCP-3/CCL7**	0.205	0.249
**IL-1β**	0.178	0.095
**uPAR**	0.143	0.168
**MIP-1δ/CCL15**	0.121	0.247
**TGF-β**	0.115	---
**I-TAC/CXCL11**	0.110	---
**M-CSF/CSF1**	0.092	0.075
**AgRP(ART)**	0.091	0.105
**EGF**	0.085	0.154
**BMP-4**	0.084	0.11
**RANTES/CCL5**	0.082	0.143
**TNF-α**	0.079	0.096
**NT-3**	0.062	---
**SPG130**	0.058	---
**IGFBP-6**	0.056	---
**BTC**	0.054	---
**ANG**	0.052	0.079
**BDNF**	0.042	---
**BLC/CXCL13**	0.042	---
**PARC/CCL18**	0.035	---
**GDNF**	0.028	---
**GITR-Light**	0.028	---
**PIGF/PGF**	0.028	---
**CKβ-8-1/CCL23**	0.026	---
**GRO-α/CXCL1**	0.026	---
**TECK/CCL25**	0.024	0.078
**MIF**	0.023	---
**FAS**	0.022	---
**MCP-1/CCL2**	0.018	---
**GRO-α,β,γ/CXCL1,2,3**	---	0.136

Supplementary Table S2. The mean SHAP scores for those above 0, for the models developed with XGBoost (36 proteins) and LightGBM (20 proteins).

International Journal of Molecular Sciences

MDPI

	Feature importance score		Feature importance score
**IL-1α**	**0.2092**	RANTES/CCL5	0.0046
**NAP-2**	**0.0568**	MSP-α/MST1	0.0043
**IL-12 p70**	**0.0547**	IL-5	0.0033
**IL-17**	**0.0454**	MIP-1α/CCL3	0.0032
**FGF-6**	**0.0423**	TNF-α	0.0032
**GRO-α/CXCL1**	**0.0384**	GM-CSF/CSF2	0.0024
**sTNF RII/TNFRSF1B**	**0.0374**	CTACK/CCL27	0.0023
**CKβ-8-1/CCL23**	**0.0373**	AXL	0.0021
**ANG-2/ANGPT-2**	**0.0336**	ANG	0.0021
**BTC**	**0.0332**	GRO-α,β,γ/CXCL1,2,3	0.0020
**MCP-4/CCL13**	**0.0315**	BMP-4	0.0019
**IGFBP-6**	**0.0295**	IL-12 p40	0.0016
**FGF-9**	**0.0290**	IGFBP-4	0.0015
**ENA-78/CXCL5**	**0.0279**	MIP-1δ/CCL15	0.0014
**MDC/CCL22**	**0.0254**	sTNF RI/TNFRSF1A	0.0014
**M-CSF/CSF1**	**0.0234**	NT-4	0.0012
**BDNF**	**0.0189**	ICAM-3	0.0010
**TGF-β**	**0.0186**	GCSF/CSF3	0.0007
**FGF-7**	**0.0130**	IL-6	0.0005
**MCP-1/CCL2**	**0.0127**	Eotaxin/CCL11	0.0005
**IL-1RA/IL1RN**	**0.0120**	TIMP-2	0.0004
**IL-2**	**0.0114**	IL-2 Ra	0.0004
**PDGF-BB**	**0.0113**	CCL-28	0.0003
**EGF**	**0.0111**	GITR-Light	0.0003
**I-TAC/CXCL11**	**0.0111**	PARC/CCL18	0.0002
**IFN-γ**	**0.0103**	SCF	0.0002
IGFBP-2	0.00997	TGF-β3	0.0001
AgRP(ART)	0.0099	DTK/TYRO3	0.0001
LIGHT/TNFSF14	0.0091	TRAIL R3/TNFRSF10C	0.0001
IL-13	0.0080	MCP-3/CCL7	0.0001
IGF-1 SR	0.0069	MCP-2/CCL8	0.0001
TIMP-1	0.0058	HCC-4/CCL16	0.0001
IL-15	0.0057	VEGF-D	0.0001
TECK/CCl25	0.0055	SDF-1/CXCL12	0.0001
GCP-2/CXCL6	0.0052	I-309/CCL1	1.985E-06
ICAM-1	0.0046	PIGF/PGF	6.317E-06

Supplementary Table S3. The feature importance scores for the model developed with TabNet with those above 0 (26 proteins with mean score ≥ 0.01, as indicated in bold).

International Journal of Molecular Sciences

MDPI

	Mean SHAP		Mean SHAP		Mean SHAP
**IL-1α**	**0.2695**	HCC-4/CCL16	0.0073	bFGF/FGF-2	0.0044
**IL-11**	**0.1191**	Eotaxin-3/CCL26	0.0071	MIP-1δ/CCL15	0.0044
**TNF-α**	**0.0832**	GITR/TNFRSF18	0.0070	Eotaxin/CCL11	0.0044
**PDGF-BB**	**0.0721**	GRO-α,β,γ/CXCL1,2,3	0.0070	MIP-1α/CCL3	0.0044
**IL-10**	**0.0688**	I-309/CCL1	0.0066	MIP-3β/CCL19	0.0043
**ANG-2/ANGPT-2**	**0.0511**	EGF-R	0.0064	AR/ARGE	0.0043
**IL-16**	**0.0445**	IL-5	0.0063	OSM	0.0043
**PIGF/PGF**	**0.0376**	GDNF	0.0063	ANG	0.0043
**FAS**	**0.0315**	BMP-6	0.0062	IL-15	0.0043
**NT-3**	**0.0302**	TIMP-2	0.0061	Eotaxin-2	0.0043
**IL-4**	**0.0269**	IFN-γ	0.0061	Acrp30/ADIPOQ	0.0042
**IL-3**	**0.0258**	LIGHT/TNFSF14	0.0058	FGF-4	0.0042
**Lymphotactin/XCL1**	**0.0256**	GM-CSF/CSF2	0.0057	MSP-α/MST1	0.0042
**M-CSF/CSF1**	**0.0247**	TNF-β	0.0056	IGF-1 SR	0.0042
**OST/TNFRSF11B**	**0.0236**	NT-4	0.0055	TRAIL R3/TNFRSF10C	0.0041
**IL-1β**	**0.0224**	TIMP-1	0.0055	MDC/CCL22	0.0041
**IL-13**	**0.0219**	ICAM-3	0.0055	IL-6 R	0.0041
**EGF**	**0.0213**	CTACK/CCL27	0.0055	TGF-β3	0.0041
**IL-1RA/IL1RN**	**0.0187**	IL-1 RI	0.0054	CCL-28	0.0041
**CKβ-8-1/CCL23**	**0.0160**	VEGF-B	0.0054	PARC/CCL18	0.0041
**GCSF/CSF3**	**0.0143**	BDNF	0.0053	IL-8/CXCL8	0.0040
**TRAIL R4/TNFRSF10D**	**0.0134**	IGFBP-4	0.0053	AgRP(ART)	0.0040
**FGF-6**	**0.0133**	FGF-9	0.0051	MCP-1/CCL2	0.0040
**I-TAC/CXCL11**	**0.0123**	AXL	0.0051	MCP-2/CCL8	0.0040
**MIP-1β/CCL4**	**0.0117**	MCP-3/CCL7	0.0050	VEGF-D	0.0040
**IL-12 p70**	**0.0104**	MIP-3α/CCL20	0.0050	FGF-7	0.0040
IL-6	0.0094	GITR-Light	0.0049	ICAM-1	0.0040
BLC/CXCL13	0.0093	IL-2 Ra	0.0047	uPAR	0.0039
BMP-4	0.0093	IGFBP-1	0.0047	MCP-4/CCL13	0.0039
LEPTIN(OB)	0.0092	SPG130	0.0047	IL-12 p40	0.0039
HGF	0.0091	NAP-2	0.0046	SCF	0.0038
IL-7	0.0089	sTNF RI/TNFRSF1A	0.0046	IGF-1	0.0038
RANTES/CCL5	0.0088	IL-2	0.0046	BTC	0.0038
DTK/TYRO3	0.0087	TGF-β	0.0046	SDF-1/CXCL12	0.0038
Fit-3 Ligand	0.0086	GRO-α/CXCL1	0.0046	IGFBP-2	0.0038
GCP-2/CXCL6	0.0086	MIG/CXCL9	0.0045	IL-17	0.0038
ENA-78/CXCL5	0.0085	Fractalkine/CX3CL1	0.0045	IGFBP-6	0.0038
IL-1R4 /ST2/IL1RL1	0.0080	β-NGF	0.0044	TECK/CCl25	0.0037
IGFBP-3	0.0078	TPO	0.0044	sTNF RII/TNFRSF1B	0.0036
CNTF	0.0075	MIF	0.0044	TARC/CCL17	0.0033

Supplementary Table S4. The mean SHAP scores for the model developed with TabPFN (26 proteins with mean score ≥ 0.01, as indicated in bold).

International Journal of Molecular Sciences

MDPI

GO ID	Term description	Strength	Signal	FDR p-val.	Proteins
GO:0050731	Positive regulation of peptidyl-tyrosine phosphorylation	1.74	2.2	7.03E-06	IL11, EGF, IL3, PDGFB, TNF, CCL5
GO:0008284	Positive regulation of cell population proliferation	1.18	1.33	1.30E-05	IL1A, IL11, EGF, IL3, CSF1, PDGFB, TNF, CCL5
GO:0030334	Regulation of cell migration	1.19	1.34	1.30E-05	IL1A,EGF, ANGPT2, CSF1, PDGFB, CCL7, TNF, CCL5
GO:0030335	Positive regulation of cell migration	1.37	1.63	1.30E-05	IL1A, EGF, CSF1, PDGFB, CCL7, TNF, CCL5
GO:0043410	Positive regulation of MAPK cascade	1.42	1.69	1.30E-05	IL1A, IL11, EGF, PDGFB, CCL7, TNF, CCL5
GO:1902533	Positive regulation of intracellular signal transduction	1.16	1.29	1.30E-05	IL1A, IL11, EGF, CSF1, PDGFB, CCL7, TNF, CCL5
GO:0045840	Positive regulation of mitotic nuclear division	2.20	2.46	1.44E-05	IL1A, EGF, PDGFB, TNF
GO:0002687	Positive regulation of leukocyte migration	1.77	2.12	1.56E-05	IL1A, CSF1, CCL7, TNF, CCL5
GO:0019221	Cytokine-mediated signaling pathway	1.46	1.71	2.03E-05	IL1A, CSF1, PDGFB, CCL7, TNF, CCL5
GO:0001934	Positive regulation of protein phosphorylation	1.22	1.34	3.21E-05	IL11, EGF, IL3, CSF1, PDGFB, TNF, CCL5
GO:0030155	Regulation of cell adhesion	1.20	1.29	4.08E-05	IL1RN, IL1A, ANGPT2, CSF1, PDGFB, TNF, CCL5
GO:0070374	Positive regulation of ERK1 and ERK2 cascade	1.62	1.78	5.51E-05	IL1A, PDGFB, CCL7, TNF, CCL5
GO:0014068	Positive regulation of phosphatidylinositol 3-kinase signaling	1.96	2.02	6.72E-05	EGF, PDGFB, TNF, CCL5
GO:0050920	Regulation of chemotaxis	1.60	1.73	6.72E-05	ANGPT2, CSF1, PDGFB, CCL7,CCL5
GO:0050900	Leukocyte migration	1.56	1.66	8.61E-05	CSF1, PDGFB, CCL7, TNF, CCL5
GO:0006954	Inflammatory response	1.30	1.35	9.49E-05	IL1RN, IL1A, CSF1, CCL7, TNF, CCL5
GO:0071675	Regulation of mononuclear cell migration	1.76	1.7	0.00021	CSF1, CCL7, TNF, CCL5
GO:0097529	Myeloid leukocyte migration	1.72	1.61	0.0003	CSF1, PDGFB, CCL7, CCL5
GO:0050921	Positive regulation of chemotaxis	1.69	1.56	0.00035	CSF1, PDGFB, CCL7, CCL5
GO:0010594	Regulation of endothelial cell migration	1.63	1.46	0.00054	EGF, ANGPT2, PDGFB, TNF
GO:0045860	Positive regulation of protein kinase activity	1.33	1.22	0.00054	EGF, CSF1, PDGFB, TNF, CCL5
GO:0002548	Monocyte chemotaxis	2.1	1.68	0.00055	PDGFB, CCL7, CCL5
GO:0010469	Regulation of signaling receptor activity	1.6	1.42	0.00063	IL1RN, EGF, TNF, CCL5
GO:0032103	Positive regulation of response to external stimulus	1.3	1.15	0.00071	CSF1, PDGFB, CCL7, TNF, CCL5
GO:0045785	Positive regulation of cell adhesion	1.27	1.10	0.00096	IL1A, CSF1, PDGFB, TNF, CCL5
GO:1900127	Positive regulation of hyaluronan biosynthetic process	2.95	1.66	0.00099	EGF, PDGFB
GO:0042531	Positive regulation of tyrosine phosphorylation of STAT protein	1.92	1.45	0.0013	IL3, TNF, CCL5
GO:0061098	Positive regulation of protein tyrosine kinase activity	1.92	1.45	0.0013	EGF, PDGFB, CCL5
GO:2000272	Negative regulation of signaling receptor activity	1.9	1.41	0.0015	IL1RN, TNF, CCL5
GO:2000503	Positive regulation of natural killer cell chemotaxis	2.78	1.54	0.0016	CCL7, CCL5
GO:0071677	Positive regulation of mononuclear cell migration	1.86	1.35	0.0019	CCL7, TNF, CCL5
GO:0046425	Regulation of receptor signaling pathway via JAK-STAT	1.84	1.33	0.0021	EGF, TNF, CCL5
GO:0002526	Acute inflammatory response	1.83	1.31	0.0022	IL1RN, IL1A, TNF
GO:0048661	Positive regulation of smooth muscle cell proliferation	1.80	1.28	0.0025	PDGFB, TNF, CCL5

GO:0043535	Regulation of blood vessel endothelial cell migration	1.78	1.25	0.0028	ANGPT2, PDGFB, TNF
GO:0002676	Regulation of chronic inflammatory response	2.55	1.37	0.0031	TNF, CCL5
GO:0002690	Positive regulation of leukocyte chemotaxis	1.73	1.19	0.0036	CSF1, CCL7, CCL5
GO:0071346	Cellular response to interferon-gamma	1.71	1.16	0.0041	CCL7, TNF, CCL5
GO:0043406	Positive regulation of MAP kinase activity	1.67	1.09	0.0053	EGF, PDGFB, TNF
GO:0048245	Eosinophil chemotaxis	2.32	1.17	0.0068	CCL7, CCL5
GO:0001936	Regulation of endothelial cell proliferation	1.59	1.00	0.0078	EGF, PDGFB, TNF
GO:0010759	Positive regulation of macrophage chemotaxis	2.28	1.13	0.0081	CSF1, CCL5
GO:0072012	Glomerulus vasculature development	2.23	1.09	0.0093	ANGPT2, PDGFB
GO:0010893	Positive regulation of steroid biosynthetic process	2.21	1.09	0.0094	IL1A, TNF
GO:0034114	Regulation of heterotypic cell-cell adhesion	2.17	1.06	0.0102	IL1RN, TNF
GO:0045672	Positive regulation of osteoclast differentiation	2.14	1.04	0.0113	CSF1, TNF

Supplementary Table S5. Enriched Gene Ontology Biological Process terms of the 13 proteins found in at least three prediction models with STRING.

KEGG ID	Term Description	Strength	Signal	FDR p-val.	Proteins
hsa04060	Cytokine-cytokine receptor interaction	1.71	3.39	1.06E-10	IL1RN, IL1A, IL11, IL3, CSF1, CCL7, TNF, CCL5
hsa04640	Hematopoietic cell lineage	2	3.15	1.20E-07	IL1A, IL11, IL3, CSF1, TNF
hsa05323	Rheumatoid arthritis	2.03	3.19	1.20E-07	IL1A, IL11, CSF1, TNF, CCL5
hsa04010	MAPK signaling pathway	1.57	2.3	3.66E-07	IL1A, EGF, ANGPT2, CSF1, PDGFB, TNF
hsa04061	Viral protein interaction with cytokine and cytokine receptor	1.87	2.24	1.35E-05	CSF1, CCL7, TNF, CCL5
hsa04151	PI3K-Akt signaling pathway	1.41	1.56	4.30E-05	EGF, IL3, ANGPT2, CSF1, PDGFB
hsa04630	JAK-STAT signaling pathway	1.66	1.79	6.66E-05	IL11, EGF, IL3, PDGFB
hsa04015	Rap1 signaling pathway	1.55	1.58	0.00015	EGF, ANGPT2, CSF1, PDGFB
hsa04014	Ras signaling pathway	1.5	1.49	0.00021	EGF, ANGPT2, CSF1, PDGFB
hsa04668	TNF signaling pathway	1.68	1.39	0.001	CSF1, TNF, CCL5
hsa04380	Osteoclast differentiation	1.65	1.34	0.0012	IL1A, CSF1, TNF
hsa05418	Fluid shear stress and atherosclerosis	1.62	1.31	0.0013	IL1A, PDGFB, TNF
hsa05164	Influenza A	1.52	1.16	0.0024	IL1A, TNF, CCL5
hsa05310	Asthma	2.12	1.35	0.0027	IL3, TNF
hsa05332	Graft-versus-host disease	2	1.22	0.0044	IL1A, TNF
hsa04940	Type I diabetes mellitus	1.97	1.21	0.0046	IL1A, TNF
hsa05321	Inflammatory bowel disease	1.78	1.02	0.0095	IL1A, TNF

Supplementary Table S6. Enriched KEGG pathway terms of the 13 proteins found in at least three prediction models with STRING.

International Journal of Molecular Sciences

MDPI

Information	Ref
**Found by all six algorithms:**	
**ANG-2/ANGPT-2**	
CSF ANG-2/ANGPT-2 levels were measured in three cohorts consisting of: 1. ATN+ AD vs. control, 2. AD vs. MCI vs. cognitively unimpaired with parental history of AD, and 3. neurologically normal, with paired serum samples. In the first cohort, CSF ANGPT-2 was increased in AD and correlated with t-tau, p-tau181, but not Aβ42, and BBB impairment markers, soluble-PDGFRβ and fibrinogen. In the second cohort, the highest ANGPT-2 levels were found in the MCI group, and correlated with t-tau, p-tau, neuronal injury markers, neurogranin and α-synuclein, and neuroinflammation markers, GFAP and YKL-40. In the third cohort, CSF ANGPT-2 was strongly correlated with the CSF/serum albumin ratio, and serum ANGPT-2 was positively correlated with CSF ANGPT-2 and CSF/serum albumin ratio, although not statistically significant. The authors thus concluded that CSF ANGPT-2 is associated with tau pathology, BBB damage, and neuronal injury in early pathogenesis, and that serum ANGPT-2 may also serve as a biomarker.	[1]
Upregulated in postmortem brain tissues from AD patients, compared to controls.	[2]
Serum ANG-2 was associated with decreased white matter fractional anisotropy specifically among APOE-ε4 carriers.	[3]
Increased crude BA7 tissue homogenate ANG-2/ANGPT-2 levels were found in AD patients with Braak stages V-VI compared to Braak stages III-IV. Crude BA7 tissue homogenate of its receptor, TIE-2 tyrosine kinase, was reduced in patients with stages III-IV, compared to 0-II and V-VI, and in enriched microvessels homogenate, levels were reduced in patients with stage V-VI compared to 0-II.	[4]
In a murine CNS autoimmune encephalomyelitis model, Ang-2 blockage with anti-Ang-2 antibody attenuated neuroinflammation, spinal cord demyelination, CNS leukocyte infiltration, and endothelial cell adhesion molecule expression, while improving BBB integrity and reducing the expression of genes relevant to antigen presentation and proinflammatory responses of microglia and macrophages, and inhibiting α5β1 integrin activation in microglia.	[5]
Ang-2 gain-of-function mice showed increased BBB permeability, with downregulation of tight/adherens junction molecules in endothelial cells and upregulation of permeability-related molecule, caveolin-1. With experimental stoke, *Ang-2* gain-of-function mice showed augmented infarct sizes and vessel permeability.	[6]
In an *APP* transgenic mouse model, increased cortical vessels and Ang-2 upregulation were found. Treatment of neural stem cell line with synthetic Aβ1–42 directly increased Ang-2 expression both at the transcript and protein level. The author suggests that Ang-2 may be one of the mediators of Aβ peptide angiogenesis regulation.	[7]
Given the reduced brain perfusion and BBB known to occur in AD, single-nucleus RNA-sequencing was performed on vascular cells from AD versus control brains – results showed that *ANG-2/ANGPT2* and other transcripts related to ANG-Tie-2 signaling were increased in endothelial cells, and validation of ANGPT2 protein expression with immunostaining also showed increased expression.	[8]
In the cerebral cortex of the murine model expressing human AβPP with the Arctic (p. E693G) and Swedish (p. KM670/671NL) mutations, which develops severe CAA and parenchymal Aβ plaques early, Ang-2, Ang-1, and Tie-2 were transcriptionally upregulated, while *Hif1α* and *Vegfr2* were downregulated.	[9]
In the presence of *Mycoplasma pulmonis* infection and thus high inflammation condition, Ang-2 binds Tie-2 in an antagonistic manner, suppressing its downstream phosphorylation, and promoting forkhead box O1 (FOXO1) activation and increased ANG-2 expression by a positive feedback loop, leading to increased pathological vascularity and vessel permeability; whereas in the absence of the pathogen, Ang-2 activates Tie-2, leading to enlarged vessels without leakiness.	[10]
**EGF**	
Low baseline plasma EGF was predictive of worse long-term cognitive outcomes in both AD and PD cohorts. For the AD cohort, amnestic MCI and AD patients' plasma EGF was reduced compared to the cognitively normal control.	[11]
Plasma EGF, PDGF-BB and MIP-1d were significantly elevated in AD, compared to controls.	[12]
Plasma EGF, GDNF, and MIP1δ were significantly elevated in AD, MIP4 in MCI, and RANTES in MCI and AD.	[13]
In two Han Chinese cohorts, a rare loss-of-function variant, and a common variant were found to be associated with AD. Further evaluation of the common variant risk allele showed that it was associated with increased *EGF* transcript expression in hippocampal tissue of postmortem AD brain.	[14]
Using a female murine model with hemizygous APP and PSEN1 mutations, and AD patient postmortem brain tissue, as well as previously published single nuclei RNA-sequencing data, EGF, Ephrin-A (EPHA), Claudin (CLDN), and Bone morphogenetic protein (BMP) signaling were identified as being predicted to have the most significant disease-related changes.	[15]
Low plasma EGF was found in female mice expressing human APOE4, which overexpresses human Aβ42 and results in cognitive impairment and cerebrovascular dysfunction. EGF treatment in these animals ameliorated cognitive decline	[16,17]

and was associated with low microbleeds and higher cerebrovascular coverage, despite no change in Aβ level.	
Treatment of single brain endothelial cell cultures and triple cultures (endothelial cells, astrocytes, and pericytes) mimicking a microvascular unit with oligomeric Aβ42 results in decreased angiogenesis and increased vessel disruption; however, EGF treatment prevented this effect, suggesting its use as a potential therapeutic for AD.	[18]
Pharmacological inhibitors of EGF-receptor (EGFR) in a *Drosophila* AD model with pan-neuronal expression of human Aβ42 or APP/PS1 murine transgenic model resulted in improved memory behavioral test outcome.	[19]
In mice, pharmacological inhibition of EGFR/HER2 with varlitinib reduced LPS- and tau-induced neuroinflammatory response, and in young tau-overexpressing mice, it inhibited the activation of microglia and astrocyte activation, and tau hyperphosphorylation.	[20]
**IL-1α**	
IL-1α and *IL-1β* polymorphisms were associated with increased AD risk, with both alleles having an enhanced risk.	[21]
Increased serum IL-1α, IL-1β, and IL-1RA were detected among AD patients compared to healthy controls, and for IL-1RA, between AD and and subjective memory complaints patients, and between MCI and subjective memory complaints patients.	[22]
In brain sections from Down syndrome and AD patients, IL-1 immunoreactive glial cells were significantly increased compared with controls, with most classified as microglia. On the other hand, IL-1 immunoreactive neuron number was not significantly changed. IL-1 was also increased in homogenates from the temporal lobe of AD patients, together with S-100 and GFAP.	[23]
In AD patient brain samples, having increased tau 2-immunoreactive neuritic plaques, activated IL-1α+ microglia and tau2+ neuritic plaques were positively correlated, as well as activated IL-1α+ microglia and activated astrocytes.	[24]
In a meta-analysis, an *IL-1α* -889C > T polymorphism was overall significantly associated with increased AD risk – this study used several previous studies, with some having shown an association, while others have not.	[25]
A strong association was found for an IL-1α polymorphism with early-onset AD.	[26]
IL-1α -889 polymorphism was associated with later-onset AD, and especially among homozygotes.	[27]
In brain samples from head trauma non-survivors, IL-1α+ microglia and βAPP+ neurons were increased, compared to controls who were non-survivors without head trauma.	[28]
Plasma IL-1α was correlated with the AD assessment scale-cognitive subscale and Aβ40, while TNF-α was correlated with the Clinical Dementia Rating.	[29]
Meta-analysis showed a significant association between *IL-1α* and AD, especially among early-onset patients.	[30]
IL-1α and IL-1β can lead to increased APP translation, and further assessment with a reporter system showed that they regulate the 5'-untranslated region (UTR) of APP	[31]
IL-1α upregulated α-disintegrin and metalloproteinase (ADAM)-10 and -17 to promote soluble amyloid precursor protein-α (sAPPα) release. IL-1α stimulation of sAPPα secretion depended on initial p38 MAPK activation, and subsequent MEK and PI3K activation.	[32]
IL-1α induces free radical nitric oxide in primary human astrocytes.	[33]
**PDGF-BB**	
Given that PDGF-BB::PDGFRβ signaling in pericytes has been well-established to be critical in healthy BBB maintenance, and BBB breakdown and pericyte loss are known to occur in AD, the study characterized its downstream signaling pathways. In primary human brain cell preparations, PDGF-B was found to be primarily expressed in endothelial cells, whereas PDGFRβ was primarily expressed in pericytes. PDGFRB was found to be transcriptionally upregulated overall in temporal cortex tissue homogenate in AD compared to control, also with increased *in situ* staining puncta in lectin-positive blood vessels, whereas PDGF-B puncta in lectin-positive blood vessels were decreased. The effect of PDGF-BB treatment in human pericyte cell culture +/- PDGFRβ inhibitor, PI3K inhibitor, and MEK/ERK inhibitor was assessed to evaluate specific mechanisms. PDGF-BB treatment was found to promote pericyte proliferation and apoptosis protection through the ERK signaling pathway, whereas PDGF-BB::PDGFRβ signaling through Akt was found to increase pericyte-derived inflammatory secretions.	[34]
Plasma EGF, PDGF-BB and MIP-1δ were significantly elevated in AD, compared to controls.	[12]
PDGF-BB staining was found in neurons, NFTs, neuropil threads, and subpopulation of neuritic plaque component, and in AD patient samples compared to control, its staining immunoreactivity correlated with neuronal loss, although increased protein expression was detected in lysate from AD frontal cortex samples compared to control.	[35]
Increased CSF soluble-PDGFRβ levels were found in MCI patients compared to control. Increased CSF soluble-PDGFRβ levels were also found in *Pdgfrβ*$^{+/-}$ and Tg2576 AD mouse models compared wild-type control, both, which are known to have significant loss of brain peritocytes.	[36]
Since PDGF-BB is established to be crucial for vasculature and BBB maintenance, circulating PDGF-BB was hypothesized to be associated with white matter hyperintensities of presumed vascular origin and small vessel damage; increased circulating PDGF-BB was indeed found to be associated with white matter hyperintensities burden in older	[37]

adults.	
Levels of PDGFRβ, the receptor for PDG-BB, were associated with ATN+ AD profile, but not with CAA or amnestic MCI clinical phenotypes.	[38]
In mice, aberrant circulating PDGF-BB was found with aging and high-fat diet challenge, and was associated with capillary reduction, pericyte loss, and increased hippocampus BBB permeability.	[39]
In a mouse model, increased bone preosteoclast PDGF-BB secretion was found to increase neuroinflammation.	[40]
Serum PDGF-BB levels in aged male mice were higher, with increased brain cerebrovascular calcification, compared to young mice. Transcriptional profiling showed that PDGF-BB upregulated genes important for osteogenic differentiation and *Slc20a1* phosphate transporter in cerebral microvessels, via promoting the phosphorylation to yield p-PDGFRβ and p-ERK, and activation of RUNX2.	[41]
PDG-BB:PDGFRβ signaling in pericytes was found to be important for BBB integrity. Deficiency in pericytes and age-related vascular damage contributed to neurodegeneration, neuroinflammation, and learning and memory loss.	[42]
**Found by five algorithms:**	
**IL-3**	
In mice, it was shown that upon Aβ deposit recognition, microglia upregulate the IL-3-specific receptor, IL-3Ra, making them responsive to IL-3 produced by astrocytes, leading to their reprogramming; the reprogrammed microglia have an acute immune response, enhanced motility, and improved ability to cluster and clear Aβ and tau aggregates, thus reducing disease pathology and cognitive decline. In human tissues, the IL3 receptor, IL3Ra was increased among APOE-ε4 genotype AD patients, correlated with increased Aβ40 and Aβ42, and disease duration.	[43]
CSF IL-3 was significantly correlated with sTREM2, Aβ42, p-tau and t-tau at baseline, and cognitive decline. Mediation pathway analysis suggested that IL-3 altered sTREM2 to contribute to tau pathology (p-tau/t-tau) and thus cognitive decline; and Aβ42 may contribute to IL-3 and TREM2 induction, to tau pathology, leading to cognitive decline.	[44]
**IL-11**	
CSF IL-11 levels were elevated in AD and FTLD patients, compared with controls, and in AD patients, correlated with MMSE cognitive scores, but not Aβ42, total-tau and p-tau.	[45]
In the rat neuroblastoma cell line, IL-11 inhibited Aβ42-induced neurotoxicity and inhibited L-phosphoserine phosphatase activation.	[46]
IL-11 prevented H_2O_2-induced neuronal cell apoptosis by activating the JAK/STAT pathway.	[47]
IL-11 stimulates neurogenesis.	[48]
It was also shown to alleviate neuropathic damage resulting from cerebral ischemia-reperfusion injury by reducing proinflammatory cytokines while increasing anti-inflammatory cytokines, limiting glial activation, and inhibiting oxidative stress and apoptosis	[49]
**M-CSF/CSF-1**	
Treatment of 5xFAD AD model mice with the PLX5622 CSF1R inhibitor, promotes microglial depletion, as CSF1R promotes microglial survival. Plaques do not form in microglial-depleted parenchyma, but only in areas with surviving microglia, and rather, Aβ deposits in cortical blood vessels similarly to CAA. In PLX5622-treated microglia-depleted animals, the hippocampal gene expression is also reversed, whereas residual plaque-forming microglia show AD-associated microglial transcriptional profile.	[50]
Treatment of primary human microglia with M-CSF resulted in proliferation, expression changes in various transcription factors, and a functional increase in Aβ42 peptide phagocytosis .	[51]
CSF1 was significantly increased in the CA4 subfield of AD patients, whereas it was decreased in CAA patients. Astrocytes with increased CSF1 levels were associated with neurotoxicity relevant to AD pathology, whereas astrocytes with reduced CSF1 expression were associated with vascular damage relevant to CAA pathology.	[52]
Microglia CSF-1 expression was upregulated in lesions of AD and amytrophic lateral sclerosis postmortem brains.	[53]
CSF-1 and CSF-1R transcripts were overexpressed in AD and MCI cases, compared to the control in inferior temporal gyrus samples, but not the cerebellum. CSF-1 and IL-34 treatment of human microglial cells resulted in the downregulation of various genes related to lysosomal function and Aβ removal.	[54]
Injection of M-CSF to APP_{Swe}/PS1 AD murine model led to an increased number of microglia, decreased Aβ deposits, and reduced the size and density of senile plaques, compared to vehicle control. Relatively more microglia-phagocytosed Aβ peptides were found in the late endosomes and lysosomes.	[55]
CSF1R inhibitors, PLX5622 and PLX3397, both caused microglial loss in cerebellar slices and adult mouse brain, *ex vivo*, and reduced oligodendrocyte progenitor cell numbers with high doses in primary cultures *in vitro* and *ex vivo*. The treatments did not have a significant impact on mature oligodendrocytes.	[56]

TNF-α	
Biologic TNF-α inhibitors, etanercept and one engineered to cross the BBB by fusion of the extracellular domain of type-II human TNF-α receptor to transferrin receptor antibody, both improved neuronal health in PS19 human tau-overexpressing mice.	[57]
Treatment of APP/PS1 mice with the fusion protein, cTfRMAb-TNFR, a biologic TNF-α inhibitor, significantly reduced brain Aβ peptide and plaque, neuroinflammatory marker ICAM-1, and a BBB disruption marker, parenchymal IgG, and improved recognition memory.	[58]
TNF-α and IL-10 polymorphisms were associated with increased p-tau CSF levels, while IL-1β and IL-6 polymorphisms were associated with increased CSF t-tau levels.	[59]
Serum IL-1β was higher in AD patients compared to controls, and TNF-α and IL-6 were inversely correlated with MMSE scores among AD patients with depression.	[60]
Plasma reduced TNF-α and increased histamine were found among early and late-onset AD patients compared to controls, and increased IL-1β was additionally found for early-onset patients. MMSE scores were positively correlated with TNF-α levels, and negatively correlated with histamine concentration.	[61]
Among this study's subjects with mild to severe AD, approximately half experienced acute systemic inflammatory events, and they were associated with increased serum TNF-α levels and increased rate of cognitive decline. High baseline TNF-α levels were also associated with faster cognitive decline, while those with low levels throughout the 6-month study period did not show cognitive decline.	[62]
CSF TNF-α and tau were significantly increased, and TGF-β and Aβ were significantly reduced in the MCI patient group compared to the control, and those who progressed to AD had significantly higher TNF-α.	[63]
Increased TNF-α was found among severe stage AD patients, compared to controls, with a decrease in CD4 lymphocytes.	[64]
TNF-α levels were decreased in the prefrontal cortex, superior temporal gyrus, and entorhinal cortex, compared to controls.	[65]
TNF-α produced from blood cells stimulated with LPS was significantly decreased from aged AD and multi-infarct dementia patients compared to no dementia controls. Circulating TNF-α was found to be increased only in multi-infract dementia patients.	[66]
Increased percentage of TNF-α, COX-2, PARP-1, CD38, C99 or presenilin-1 positive PBMCs, as well as increased plasma TNF-α were found in AD patients' samples, compared to controls.	[67]
Serum TNF-α levels were increased, and IGF-I levels were decreased among AD patients, compared to controls.	[68]
Increased spontaneous and IL-2-induced IFN-γ and TNF-α were found from NK cells in patients with dementia of AD type, compared controls, whereas serum levels were not significantly different. Spontaneous release of IFN-γ and TNF-α from NK were negatively associated with MMSE cognitive function score among patients with dementia of AD type.	[69]
Brain stereotaxic injection of TNF-α in APP/PS1 and 5xFAD murine models, led to accumulated p62 recruitment of RIPK1 and downstream signaling cascade, which led to neuronal necroptosis. In AD patients' brain tissues, p62 was upregulated, compared to control. Neuronal ell culture also showed that TNF-α treatment leads to necroptosis.	[70]
Plasma TNF-α and Aβ42 levels were higher among AD patients, compared to healthy controls. Among subjective cognitive impairment patients, TNF-α was also higher than healthy controls, and negatively correlated with Aβ42. In human microglial cell culture, TNF-α did not alter the amyloidogenic pathway, suggesting that TNF-α may be a reflection of AD conditions, rather than a contributor to AD pathogenesis.	[71]
Plasma TNF-α was correlated with the Clinical Dementia Rating, IL-1α was correlated with the AD assessment scale-cognitive subscale and Aβ40.	[29]
In the 5XFAD/Tg197 AD/TNF model mice that develop amyloid deposits and inflammatory arthritis, by human TNF-α expression, systemic treatment with the anti-human-TNF-α antibody, infliximab, which does not cross the BBB, and is known to prevent arthritis, increased amyloid deposition, rescued neuronal impairment, and ameliorated gliosis and recruitment of BBB immune cells, without impacting brain human TNF-α levels.	[72]
**Found by four algorithms:**	
**IL-1RA/IL1RN**	
Increased serum IL-1α, IL-1β, and IL-1RA were detected among AD patients compared to healthy controls, and for IL-1RA, between AD and subjective memory complaints patients, and between MCI and subjective memory complaints patients.	[22]
In 5xFAD murine AD model, aspirin increases IL-1a transcript and protein expression of IL-1Ra in primary murine astrocytes, microglial cell line, and in the cortex of mice. *IL-1Ra* was transcriptionally regulated by PPARα.	[73]
In aged mice, augmented hippocampal IL-1β post-surgery was associated with cognitive dysfunction, and intracisternal IL-1RA treatment alleviated this effect.	[74]
In mice, LPS treatment caused elevated plasma TNF-α, increased IL-1β transcript in the hippocampus, and microgliosis	[75]

associated with increased HMGB-1 and memory dysfunction – IL-1Ra alleviated the plasma cytokine and memory dysfunction, although it did not impact HMGB-1 levels.	
Rats injected with BCG have increased hippocampal IL-1β and spatial memory impairment, while IL-1Ra initially increased, but declined after. Treatment with IL-1Ra alleviated memory decline.	[76]
**Found by three algorithms:**	
**CKβ-8-1/CCL23**	
Plasma and CSF CCL23 were associated with MCI cases that progressed to AD; and APOEε4 allele was associated with elevated CCL23.	[77]
**G-CSF/CSF3**	
Significantly reduced CSF levels for CSF3 were found for APOE4 patients compared to APOE3.	[78]
Plasma G-CSF, known to promote neuroprotective effects and promote neurogenesis in the brain, was reduced in AD patients compared to control, and was inversely correlated with CSF Aβ1-42 and age, but not to tau or MMSE scores.	[79]
In the Tg-APP/PS1 murine model of AD, treatment with G-CSF significantly enhanced cognitive performance, decreased hippocampal and entorhinal cortex Aβ deposits, and increased microglial activity.	[80]
In rats, intracerebroventicular injection of Aβ restuls in spacial memory dysfunction, which was alleviated by GCSF treatment. This effect was associated with reduced lipid peroxidation and acetylcholinesterase levels, while antioxidant anzymes, total RNA expression, progenitor cells, and CD34+ cells indicating neurogenesis increased.	[81]
**IL-1β**	
IL-1α and *IL-1β* polymorphisms were associated with increased AD risk, with both alleles having an enhanced risk.	[21]
Increased serum IL-1α, IL-1β, and IL-1RA were detected among AD patients compared to healthy controls, and for IL-1RA, between AD and and subjective memory complaints patients, and between MCI and subjective memory complaints patients.	[22]
IL-1β and IL-6 polymorphisms were associated with increased CSF t-tau levels, while TNF-α and IL-10 polymorphisms were associated with increased p-tau CSF levels.	[59]
Serum IL-1β was higher in AD patients compared to controls, and TNF-α and IL-6 were inversely correlated with MMSE scores among AD patients with depression.	[60]
Plasma reduced TNF-α and increased histamine were found among early and late-onset AD patients compared to controls, and increased IL-1β was additionally found for early-onset patients. MMSE scores were positively correlated with TNF-α levels, and negatively correlated with histamine concentration.	[61]
In aged mice, augmented hippocampal IL-1β post-surgery was associated with cognitive dysfunction, and intracisternal IL-1RA treatment alleviated this effect.	[74]
In mice, LPS treatment caused elevated plasma TNF-α, increased IL-1β transcript in the hippocampus, and microgliosis associated with increased HMGB-1 and memory dysfunction – IL-1Ra alleviated the plasma cytokine and memory dysfunction, although it did not impact HMGB-1 levels.	[75]
Rats injected with BCG have increased hippocampal IL-1β and spatial memory impairment, while IL-1Ra initially increased, but declined after. Treatment with IL-1Ra alleviated memory decline.	[76]
IL-1β polymorphisms were associated with increased AD risk.	[82]
Among AD patients, *IL-1β* polymorphisms were associated with structural covariance in the anterior brain network and entorhinal-interconnected network, and this effect, unrelated to white matter tract integrity.	[83]
In mice, LPS treatment and surgical intervention increased IL-1β both in plasma and hippocampus, and reduced contextual freezing time, showing memory impairment.	[84]
In APP/PS1 transgenic mice, LPS stimulation resulted in increased microglia IL-1β production and increased astrocyte IL-6 response to IL-1β elevation, compared to wild-type control mice. APP/PS1 mice were also more susceptible to disrupted hippocampal gamma oscillations with IL-1β treatment and cognitive dysfunction due to LPS treatment, compared to control mice. In AD patients' postmortem brain samples, IL-1β and IL-6 were increased among those with infection compared to those without infection.	[85]
APP/PS1 mice with hippocampal IL-1β overexpression showed increased microglial proliferation and reduced Aβ and GFAP expression. Transcriptome profiling showed changes in gene expression related to immune responses, proliferation, and cytokine signaling.	[86]
Injection of synthetic IL-1β in the cerebral hemispheres of rats increased expression of the S100β astrocyte marker and two β-APP isoforms. C6 glioma cells treated with IL-1β or IL-1α resulted in increased S100β expression.	[87]
In mice, intracerebroventicular injection of Aβ oligomers activated glial cells and increased pro-inflammatory cytokine production – treatment with anti-inflammatory drug, indomethacin and IL-1β receptor agonist prevented the effect of Aβ oligomers.	[88]
In 3xTg-AD mice, treatment with an IL-1 receptor antibody significantly ameliorated cognitive deficits and reduced tau	[89]

pathology, reduced certain forms of Aβ, and reduced signaling pathway activity, including NF-kB, tau kinases (cdk5/p25, GSK-3β, p38-MAPK), p-tau levels, astrocyte marker, S100B, and neuronal Wnt/β-catenin. Treatment or primary astrocytes with IL-1β induced S100B and regulated β-catenin signaling.	
In 3xTgAD mouse background with an inducible IL-1β overexpression transgene, increased p-tau and tau-kinases, . increased p38 MAPK and GSK-3β activity, were found, yet with a significant reduction in amyloid load, and a significant increase in plaque-associated microglia and microglial activation.	[90]
In primary cultures of rat hippocampal neurons, IL-1β treatment induced NMDA receptor and tyrosine kinase activity, and NMDA-mediated neuronal cell death.	[91]
In a murine model overexpressing mutated human APP and having increased Aβ burden, TNF-α, IL-1β, and GFAP were upregulated in the microglia and localized with Aβ.	[92]
**I-TAC/CXCL11**	
Plasma CXCL11 and CCL13 were reduced and IL-6 was elevated in late-life depression, compared to amnestic MCI patients.	[93]
CSF CXCL11 was significantly elevated in AD and MCI groups, compared to the control patient group; and between AD and MCI groups, it was higher in the MCI group. CSF CXCL11 was also significantly correlated with Tau and p-Tau-181, and NGAL, but not to Aβ42 or Aβ42/40. AUROC showed that it could distinguish between AD vs. control.	[94]

Supplementary Table S7. Literature review of the 13 proteins found in at least three prediction models. [Abbreviations: Alzheimer's disease (AD), amyloid precursor protein (APP), Amyloid-Tau-Neurodegeneration (ATN), blood-brain barrier (BBB), cerebral amyloid angiopathy (CAA), central nervous system (CNS), cerebrospinal fluid (CSF), frontotemporal lobar degeneration (FTLD), mild cognitive impairment (MCI), Mini-Mental State Examination (MMSE), neurofibrillary tangles (NFTs), Parkinson's disease (PD)]

International Journal of Molecular Sciences

MDPI

**Symbol**	**ENTREZ ID**	**In the AD models developed?**	**In previous aging studies or database?**
ADIPOQ/Acrp30	9370		
AgRP(ART)	181	XGBoost, LGBM	Tanaka, Johnson, Lehallier, Coenen, Argentieri
ANG	283	XGBoost, LGBM	
ANG-2/ANGPT-2	285	EBlasso, EBEN, XGBoost, LGBM, TabNet, TabPFN	Argentieri
AR/ARGE	374		
AXL	558		CellAge
BDNF	627	XGBoost, TabNet	Lehallier, GenAge
bFGF/FGF-2	2247		CellSig
BLC/CXCL13	10563	EBEN, XGBoost	Lehallier
BMP-4	652	XGBoost, LGBM	CellAge
BMP-6	654		
β-NGF	4803		GenAge
BTC	685	XGBoost, TabNet	CellSig
CCL-28	56477		Lehallier, Argentieri
CKβ-8-1/CCL23	6368	XGBoost, TabNet, TabPFN	Lehallier, Coenen
CNTF	1270		
CTACK/CCL27	10850		Horvath
DTK/TYRO3	7301		
EGF	1950	EBlasso, EBEN, XGBoost, LGBM, TabNet, TabPFN	Tanaka, Johnson, Lehallier, GenAge
EGF-R	1956		Tanaka, Johnson, Lehallier, Coenen, Argentieri, GenAge, CellAge
ENA-78/CXCL5	6374	TabNet	Lehallier
Eotaxin/CCL11	6356		Lehallier
Eotaxin-2/CCL24	6369		
Eotaxin-3/CCL26	10344		Lehallier
FAS	355	XGBoost, TabPFN	Tanaka, Johnson, Lehallier, Coenen, Argentieri, GenAge, CellSig
FGF-4	2249		
FGF-6	2251	TabNet, TabPFN	Lehallier
FGF-7	2252	TabNet	Lehallier
FGF-9	2254	TabNet	Lehallier
Fit-3 Ligand	2323		
Fractalkine/CX3CL1	6376		Lehallier
GCP-2/CXCL6	6372		
G-CSF/CSF3	1440	XGBoost, LGBM, TabPFN	
GDNF	2668	XGBoost	
GITR/TNFRSF18	8784		
GITR-Light	8995	XGBoost	
GM-CSF/CSF2	1437		
GRO-α,β,γ/CXCL1,2,3	2919, 2920, 2921	LGBM	Tanaka, Lehallier
GRO-α/CXCL1	2919	XGBoost, TabNet	Tanaka, Lehallier, CellAge
HCC-4/CCL16	6360		Tanaka, Lehallier
HGF	3082		Johnson, Lehallier
I-309/CCL1	6346		
ICAM-1	3383		CellSig
ICAM-3	3385		
IFN-γ	3458	TabNet	CellAge

International Journal of *Molecular Sciences*

MDPI

IGF-1	3479		Coenen, GenAge
IGF-1 SR	3480		GenAge
IGFBP-1	3484		Lehallier, CellAge
IGFBP-2	3485		Lehallier, Coenen, GenAge
IGFBP-3	3486		Tanaka, Lehallier, Coenen, GenAge, CellAge, CellSig
IGFBP-4	3487		
IGFBP-6	3489	XGBoost, TabNet	Johnson, Lehallier
IL-1 RI	3554		CellAge
IL-10	3586	TabPFN	
IL-11	3589	EBlasso, EBEN, XGBoost, LGBM, TabPFN	CellSig
IL-12 p40	3593		
IL-12 p70	3592	TabNet, TabPFN	
IL-13	3596	TabPFN	Lehallier
IL-15	3600		
IL-16	3603	TabPFN	Lehallier
IL-17	3605	TabNet	
IL-1α	3552	EBlasso, EBEN, XGBoost, LGBM, TabNet, TabPFN	CellAge
IL-1β	3553	XGBoost, LGBM, TabPFN	
IL-1R4 /ST2/IL1RL1	9173		Lehallier
IL-1RA/IL1RN	3557	XGBoost, LGBM, TabNet, TabPFN	Lehallier, CellAge
IL-2	3558	TabNet	Lehallier, GenAge
IL-2 Ra	3559		
IL-3	3562	EBlasso, EBEN, XGBoost, LGBM, TabPFN	
IL-4	3565	TabPFN	
IL-5	3567		
IL-6	3569		Lehallier, GenAge, CellAge
IL-6 R	3570		
IL-7	3574		GenAge
IL-8/CXCL8	3576		Lehallier, Coenen
I-TAC/CXCL11	6373	XGBoost, TabNet, TabPFN	Johnson, Lehallier
LEPTIN(OB)	3952		Lehallier, GenAge
LIGHT/TNFSF14	8740		Lehallier
Lymphotactin/XCL1	6375	TabPFN	Argentieri
MCP-1/CCL2	6347	XGBoost, TabNet	Lehallier, CellAge
MCP-2/CCL8	6355		
MCP-3/CCL7	6354	XGBoost, LGBM	Horvath, Lehallier
MCP-4/CCL13	6357	TabNet	
M-CSF/CSF1	1435	EBEN, XGBoost, LGBM, TabNet, TabPFN	Lehallier
MDC/CCL22	6367	TabNet	Lehallier
MIF	4282	XGBoost	Tanaka, GenAge, CellAge
MIG/CXCL9	4283		Johnson, Lehallier, Argentieri
MIP-1α/CCL3	6348		Lehallier
MIP-1β/CCL4	6351	TabPFN	
MIP-1δ/CCL15	6359	XGBoost, LGBM	Lehallier
MIP-3α/CCL20	6364		Duran
MIP-3β/CCL19	6363		
MSP-α/MST1	4485		CellAge
NAP-2/CXCL7	5473	TabNet	Johnson, Lehallier
NT-3	4908	XGBoost, TabPFN	Tanaka, Argentieri
NT-4	4909		Argentieri
OSM	5008		

International Journal of
Molecular Sciences

MDPI

Osteoprotogerin/TNFRSF11B	4982	TabPFN	Johnson, Lehallier, Coenen, Argentieri
PARC/CCL18	6362	XGBoost	Tanaka
PDGF-BB	5155	EBlasso, EBEN, XGBoost, LGBM, TabNet, TabPFN	Horvath, Johnson, Lehallier, GenAge, CellAge
PIGF/PGF	5228	XGBoost, TabPFN	
RANTES/CCL5	6352	XGBoost, LGBM	Lehallier
SCF	4254		Lehallier, CellSig
SDF-1/CXCL12	6387		Argentieri, CellSig
SPG130	3572	XGBoost	Horvath
sTNF RI/TNFRSF1A	7132		Johnson, Lehallier, Coenen
sTNF RII/TNFRSF1B	8764	TabNet	
TARC/CCL17	6361		Lehallier
TECK/CCL25	6370	XGBoost, LGBM	Lehallier
TGF-β1	7040	XGBoost, TabNet	Lehallier, GenAge
TGF-β3	7043		
TIMP-1	7076		Lehallier
TIMP-2	7077		Lehallier, CellSig
TNF-α	7124	EBlasso, EBEN, XGBoost, LGBM, TabPFN	GenAge
TNF-β	4049		Tanaka, Lehallier
TPO	7066		
TRAIL R3/TNFRSF10C	8794		
TRAIL R4/TNFRSF10D	8793	TabPFN	CellSig
uPAR	5329	XGBoost, LGBM	Johnson, Lehallier, Coenen
VEGF-B	7423		
VEGF-D	2277		Lehallier, Coenen

Supplementary Table S8. Overlaps of the AD models developed (EBlasso, EBEN, XGBoost, LightGBM, TabNet, and TabPFN), with various previously published aging biomarker models or described as aging-related in those studies (DNA methylome-based (Horvath, 2013; Hannum et al., 2013; Levine et al., 2018; Belsky et al., 2022); transcriptome-based (Duran and Tsurumi, 2025); and protein-based (Tanaka et al., 2018; Lehallier et al., 2019; Johnson et al., 2020; Coenen et al., 2023; Argentieri et al., 2024); and the GenAge, CellAge and cell senescence signatures from the Human Ageing Genomic Resources (HAGR) database (Tacutu et al., 2018; de Magalhães et al., 2024).

International Journal of *Molecular Sciences*

MDPI

Supplementary References

1. Van Hulle, C.; Ince, S.; Okonkwo, O.C.; Bendlin, B.B.; Johnson, S.C.; Carlsson, C.M.; Asthana, S.; Love, S.; Blennow, K.; Zetterberg, H.; et al. Elevated CSF Angiopoietin-2 Correlates with Blood-Brain Barrier Leakiness and Markers of Neuronal Injury in Early Alzheimer's Disease. *Transl Psychiatry* **2024**, *14*, 3, doi:10.1038/s41398-023-02706-w.
2. Duche, A.H.; Tan, O.; Baskys, A.; Sumbria, R.K.; Roosan, M.R. Predictive Gene Expression Signatures for Alzheimer's Disease Using Post-Mortem Brain Tissue. *Front. Aging Neurosci.* **2025**, *17*, doi:10.3389/fnagi.2025.1591946.
3. Raman, M.R.; Himali, J.J.; Conner, S.C.; DeCarli, C.; Vasan, R.S.; Beiser, A.S.; Seshadri, S.; Maillard, P.; Satizabal, C.L. Circulating Vascular Growth Factors and Magnetic Resonance Imaging Markers of Small Vessel Disease and Atrophy in Middle-Aged Adults. *Stroke* **2018**, *49*, 2227–2229, doi:10.1161/STROKEAHA.118.022613.
4. Ince, S.; Love, S.; Minors, J.S. Dysregulated Angiopoietin-Tie Signalling Contributes to Neurovascular Dysfunction in Alzheimer's Disease. *Alzheimer's & Dementia* **2025**, *20*, e095139, doi:doi.org/10.1002/alz.095139.
5. Li, Z.; Korhonen, E.A.; Merlini, A.; Strauss, J.; Wihuri, E.; Nurmi, H.; Antila, S.; Paech, J.; Deutsch, U.; Engelhardt, B.; et al. Angiopoietin-2 Blockade Ameliorates Autoimmune Neuroinflammation by Inhibiting Leukocyte Recruitment into the CNS. *J Clin Invest* **2020**, *130*, 1977–1990, doi:10.1172/JCI130308.
6. Gurnik, S.; Devraj, K.; Macas, J.; Yamaji, M.; Starke, J.; Scholz, A.; Sommer, K.; Di Tacchio, M.; Vutukuri, R.; Beck, H.; et al. Angiopoietin-2-Induced Blood–Brain Barrier Compromise and Increased Stroke Size Are Rescued by VE-PTP-Dependent Restoration of Tie2 Signaling. *Acta Neuropathol* **2016**, *131*, 753–773, doi:10.1007/s00401-016-1551-3.
7. Sheikh, A.M.; Yano, S.; Tabassum, S.; Mitaki, S.; Michikawa, M.; Nagai, A. Alzheimer's Amyloid β Peptide Induces Angiogenesis in an Alzheimer's Disease Model Mouse through Placental Growth Factor and Angiopoietin 2 Expressions. *International Journal of Molecular Sciences* **2023**, *24*, 4510, doi:10.3390/ijms24054510.
8. Tsartsalis, S.; Sleven, H.; Fancy, N.; Wessely, F.; Smith, A.M.; Willumsen, N.; Cheung, T.K.D.; Rokicki, M.J.; Chau, V.; Ifie, E.; et al. A Single Nuclear Transcriptomic Characterisation of Mechanisms Responsible for Impaired Angiogenesis and Blood-Brain Barrier Function in Alzheimer's Disease. *Nat Commun* **2024**, *15*, 2243, doi:10.1038/s41467-024-46630-z.
9. Skaaraas, G.H.E.S.; Melbye, C.; Puchades, M.A.; Leung, D.S.Y.; Jacobsen, Ø.; Rao, S.B.; Ottersen, O.P.; Leergaard, T.B.; Torp, R. Cerebral Amyloid Angiopathy in a Mouse Model of Alzheimer's Disease Associates with Upregulated Angiopoietin and Downregulated Hypoxia-Inducible Factor. *J Alzheimers Dis* **2021**, *83*, 1651–1663, doi:10.3233/JAD-210571.
10. Kim, M.; Allen, B.; Korhonen, E.A.; Nitschké, M.; Yang, H.W.; Baluk, P.; Saharinen, P.; Alitalo, K.; Daly, C.; Thurston, G.; et al. Opposing Actions of Angiopoietin-2 on Tie2 Signaling and FOXO1 Activation. *J Clin Invest* **2016**, *126*, 3511–3525, doi:10.1172/JCI84871.
11. Lim, N.S.; Swanson, C.R.; Cherng, H.; Unger, T.L.; Xie, S.X.; Weintraub, D.; Marek, K.; Stern, M.B.; Siderowf, A.; Trojanowski, J.Q.; et al. Plasma EGF and Cognitive Decline in Parkinson's Disease and Alzheimer's Disease. *Ann Clin Transl Neurol* **2016**, *3*, 346–355, doi:10.1002/acn3.299.
12. Björkqvist, M.; Ohlsson, M.; Minthon, L.; Hansson, O. Evaluation of a Previously Suggested Plasma Biomarker Panel to Identify Alzheimer's Disease. *PLoS One* **2012**, *7*, e29868, doi:10.1371/journal.pone.0029868.
13. Marksteiner, J.; Kemmler, G.; Weiss, E.M.; Knaus, G.; Ullrich, C.; Mechtcheriakov, S.; Oberbauer, H.; Auffinger, S.; Hinterholzl, J.; Hinterhuber, H.; et al. Five out of 16 Plasma Signaling Proteins Are Enhanced in Plasma of Patients with Mild Cognitive Impairment and Alzheimer's Disease. *Neurobiol Aging* **2011**, *32*, 539–540, doi:10.1016/j.neurobiolaging.2009.03.011.
14. Li, X.; Xu, M.; Bi, R.; Tan, L.-W.; Yao, Y.-G.; Zhang, D.-F. Common and Rare Variants of EGF Increase the Genetic Risk of Alzheimer's Disease as Revealed by Targeted Sequencing of Growth Factors in Han Chinese. *Neurobiol Aging* **2023**, *123*, 170–181, doi:10.1016/j.neurobiolaging.2022.10.009.
15. Bartas, K.; Nguyen, M.; Zhao, W.; Hui, M.; Nie, Q.; Beier, K.T. Analysis of Changes in Intercellular Communications in Alzheimer's Disease Reveals Conserved Changes in Glutamatergic Transmission in Mice and Humans. *Sci Rep* **2025**, *15*, 26248, doi:10.1038/s41598-025-10795-4.
16. Thomas, R.; Zuchowska, P.; Morris, A.W.J.; Marottoli, F.M.; Sunny, S.; Deaton, R.; Gann, P.H.; Tai, L.M. Epidermal Growth Factor Prevents APOE4 and Amyloid-Beta-Induced Cognitive and Cerebrovascular Deficits in Female Mice. *Acta Neuropathol Commun* **2016**, *4*, 111, doi:10.1186/s40478-016-0387-3.

17. Thomas, R.; Morris, A.W.J.; Tai, L.M. Epidermal Growth Factor Prevents *APOE4*-Induced Cognitive and Cerebrovascular Deficits in Female Mice. *Heliyon* **2017**, *3*, e00319, doi:10.1016/j.heliyon.2017.e00319.
18. Koster, K.P.; Thomas, R.; Morris, A.W.; Tai, L.M. Epidermal Growth Factor Prevents Oligomeric Amyloid-β Induced Angiogenesis Deficits in Vitro. *J Cereb Blood Flow Metab* **2016**, *36*, 1865–1871, doi:10.1177/0271678X16669956.
19. Wang, L.; Chiang, H.-C.; Wu, W.; Liang, B.; Xie, Z.; Yao, X.; Ma, W.; Du, S.; Zhong, Y. Epidermal Growth Factor Receptor Is a Preferred Target for Treating Amyloid-β–Induced Memory Loss. *Proceedings of the National Academy of Sciences* **2012**, *109*, 16743–16748, doi:10.1073/pnas.1208011109.
20. Kim, J.; Kim, S.-J.; Jeong, H.-R.; Park, J.-H.; Moon, M.; Hoe, H.-S. Inhibiting EGFR/HER-2 Ameliorates Neuroinflammatory Responses and the Early Stage of Tau Pathology through DYRK1A. *Front. Immunol.* **2022**, *13*, doi:10.3389/fimmu.2022.903309.
21. Nicoll, J.A.; Mrak, R.E.; Graham, D.I.; Stewart, J.; Wilcock, G.; MacGowan, S.; Esiri, M.M.; Murray, L.S.; Dewar, D.; Love, S.; et al. Association of Interleukin-1 Gene Polymorphisms with Alzheimer's Disease. *Ann Neurol* **2000**, *47*, 365–368.
22. Italiani, P.; Puxeddu, I.; Napoletano, S.; Scala, E.; Melillo, D.; Manocchio, S.; Angiolillo, A.; Migliorini, P.; Boraschi, D.; Vitale, E.; et al. Circulating Levels of IL-1 Family Cytokines and Receptors in Alzheimer's Disease: New Markers of Disease Progression? *Journal of Neuroinflammation* **2018**, *15*, 342, doi:10.1186/s12974-018-1376-1.
23. Griffin, W.S.; Stanley, L.C.; Ling, C.; White, L.; MacLeod, V.; Perrot, L.J.; White, C.L.; Araoz, C. Brain Interleukin 1 and S-100 Immunoreactivity Are Elevated in Down Syndrome and Alzheimer Disease. *Proc Natl Acad Sci U S A* **1989**, *86*, 7611–7615, doi:10.1073/pnas.86.19.7611.
24. Sheng, J.G.; Mrak, R.E.; Griffin, W.S. Microglial Interleukin-1 Alpha Expression in Brain Regions in Alzheimer's Disease: Correlation with Neuritic Plaque Distribution. *Neuropathol Appl Neurobiol* **1995**, *21*, 290–301, doi:10.1111/j.1365-2990.1995.tb01063.x.
25. Mun, M.-J.; Kim, J.-H.; Choi, J.-Y.; Jang, W.-C. Genetic Polymorphisms of Interleukin Genes and the Risk of Alzheimer's Disease: An Update Meta-Analysis. *Meta Gene* **2016**, *8*, 1–10, doi:10.1016/j.mgene.2016.01.001.
26. Grimaldi, L.M.; Casadei, V.M.; Ferri, C.; Veglia, F.; Licastro, F.; Annoni, G.; Biunno, I.; De Bellis, G.; Sorbi, S.; Mariani, C.; et al. Association of Early-Onset Alzheimer's Disease with an Interleukin-1alpha Gene Polymorphism. *Ann Neurol* **2000**, *47*, 361–365.
27. Du, Y.; Dodel, R.C.; Eastwood, B.J.; Bales, K.R.; Gao, F.; Lohmüller, F.; Müller, U.; Kurz, A.; Zimmer, R.; Evans, R.M.; et al. Association of an Interleukin 1 Alpha Polymorphism with Alzheimer's Disease. *Neurology* **2000**, *55*, 480–483, doi:10.1212/wnl.55.4.480.
28. Griffin, W.S.T.; Shenga, J.G.; Gentleman, S.M.; Graham, D.I.; Mrak, R.E.; Roberts, G.W. Microglial Interleukin-1α Expression in Human Head Injury: Correlations with Neuronal and Neuritic β-Amyloid Precursor Protein Expression. *Neurosci Lett* **1994**, *176*, 133–136, doi:10.1016/0304-3940(94)90066-3.
29. Mahdavi, M.; Karima, S.; Rajaei, S.; Aghamolaii, V.; Ghahremani, H.; Ataei, R.; Tehrani, H.S.; Baram, S.M.; Tafakhori, A.; Safarpour Lima, B.; et al. Plasma Cytokines Profile in Subjects with Alzheimer's Disease: Interleukin 1 Alpha as a Candidate for Target Therapy. *Galen Med J* **2021**, *10*, e1974, doi:10.31661/gmj.v10i0.1974.
30. Rainero, I.; Bo, M.; Ferrero, M.; Valfrè, W.; Vaula, G.; Pinessi, L. Association between the Interleukin-1α Gene and Alzheimer's Disease: A Meta-Analysis. *Neurobiology of Aging* **2004**, *25*, 1293–1298, doi:10.1016/j.neurobiolaging.2004.02.011.
31. Rogers, J.T.; Leiter, L.M.; McPhee, J.; Cahill, C.M.; Zhan, S.-S.; Potter, H.; Nilsson, L.N.G. Translation of the Alzheimer Amyloid Precursor Protein mRNA Is Up-Regulated by Interleukin-1 through 5′-Untranslated Region Sequences *. *Journal of Biological Chemistry* **1999**, *274*, 6421–6431, doi:10.1074/jbc.274.10.6421.
32. Bandyopadhyay, S.; Hartley, D.M.; Cahill, C.M.; Lahiri, D.K.; Chattopadhyay, N.; Rogers, J.T. Interleukin-1α Stimulates Non-Amyloidogenic Pathway by α-Secretase (ADAM-10 and ADAM-17) Cleavage of APP in Human Astrocytic Cells Involving P38 MAP Kinase. *Journal of Neuroscience Research* **2006**, *84*, 106–118, doi:10.1002/jnr.20864.
33. Chao, C.C.; Hu, S.; Sheng, W.S.; Bu, D.; Bukrinsky, M.I.; Peterson, P.K. Cytokine-Stimulated Astrocytes Damage Human Neurons via a Nitric Oxide Mechanism. *Glia* **1996**, *16*, 276–284, doi:10.1002/(SICI)1098-1136(199603)16:3%3C276::AID-GLIA10%3E3.0.CO;2-X.
34. Smyth, L.C.D.; Highet, B.; Jansson, D.; Wu, J.; Rustenhoven, J.; Aalderink, M.; Tan, A.; Li, S.; Johnson, R.; Coppieters, N.; et al. Characterisation of PDGF-BB:PDGFRβ Signalling Pathways in Human Brain Pericytes: Evidence of Disruption in Alzheimer's Disease. *Commun Biol* **2022**, *5*, 1–16, doi:10.1038/s42003-022-03180-8.

35. Masliah, E.; Mallory, M.; Alford, M.; Deteresa, R.; Saitoh, T. PDGF Is Associated with Neuronal and Glial Alterations of Alzheimer's Disease. *Neurobiol Aging* **1995**, *16*, 549–556, doi:10.1016/0197-4580(95)00050-o.
36. Montagne, A.; Barnes, S.R.; Sweeney, M.D.; Halliday, M.R.; Sagare, A.P.; Zhao, Z.; Toga, A.W.; Jacobs, R.E.; Liu, C.Y.; Amezcua, L.; et al. Blood-Brain Barrier Breakdown in the Aging Human Hippocampus. *Neuron* **2015**, *85*, 296–302, doi:10.1016/j.neuron.2014.12.032.
37. Kapoor, A.; Nation, D.A. Platelet-Derived Growth Factor-BB and White Matter Hyperintensity Burden in *APOE4* Carriers. *Cerebral Circulation - Cognition and Behavior* **2022**, *3*, 100131, doi:10.1016/j.cccb.2022.100131.
38. De Kort, A.M.; Kuiperij, H.B.; Kersten, I.; Versleijen, A.A.M.; Schreuder, F.H.B.M.; Van Nostrand, W.E.; Greenberg, S.M.; Klijn, C.J.M.; Claassen, J.A.H.R.; Verbeek, M.M. Normal Cerebrospinal Fluid Concentrations of PDGFRβ in Patients with Cerebral Amyloid Angiopathy and Alzheimer's Disease. *Alzheimers Dement* **2022**, *18*, 1788–1796, doi:10.1002/alz.12506.
39. Liu, G.; Wang, J.; Wei, Z.; Fang, C.-L.; Shen, K.; Qian, C.; Qi, C.; Li, T.; Gao, P.; Wong, P.C.; et al. Elevated PDGF-BB from Bone Impairs Hippocampal Vasculature by Inducing PDGFRβ Shedding from Pericytes. *Advanced Science* **2023**, *10*, 2206938, doi:10.1002/advs.202206938.
40. Liu, G.; Shu, W.; Chen, Y.; Fu, Y.; Fang, S.; Zheng, H.; Cheng, W.; Lin, Q.; Hu, Y.; Jiang, N.; et al. Bone-Derived PDGF-BB Enhances Hippocampal Non-Specific Transcytosis through Microglia-Endothelial Crosstalk in HFD-Induced Metabolic Syndrome. *Journal of Neuroinflammation* **2024**, *21*, 111, doi:10.1186/s12974-024-03097-5.
41. Wang, J.; Fang, C.-L.; Noller, K.; Wei, Z.; Liu, G.; Shen, K.; Song, K.; Cao, X.; Wan, M. Bone-Derived PDGF-BB Drives Brain Vascular Calcification in Male Mice. *J Clin Invest* **2023**, *133*, e168447, doi:10.1172/JCI168447.
42. Bell, R.D.; Winkler, E.A.; Sagare, A.P.; Singh, I.; LaRue, B.; Deane, R.; Zlokovic, B.V. Pericytes Control Key Neurovascular Functions and Neuronal Phenotype in the Adult Brain and during Brain Aging. *Neuron* **2010**, *68*, 409–427, doi:10.1016/j.neuron.2010.09.043.
43. McAlpine, C.S.; Park, J.; Griciuc, A.; Kim, E.; Choi, S.H.; Iwamoto, Y.; Kiss, M.G.; Christie, K.A.; Vinegoni, C.; Poller, W.C.; et al. Astrocytic Interleukin-3 Programs Microglia and Limits Alzheimer's Disease. *Nature* **2021**, *595*, 701–706, doi:10.1038/s41586-021-03734-6.
44. Wang, Z.-B.; Ma, Y.-H.; Sun, Y.; Tan, L.; Wang, H.-F.; Yu, J.-T. Interleukin-3 Is Associated with sTREM2 and Mediates the Correlation between Amyloid-β and Tau Pathology in Alzheimer's Disease. *J Neuroinflammation* **2022**, *19*, 316, doi:10.1186/s12974-022-02679-5.
45. Galimberti, D.; Venturelli, E.; Fenoglio, C.; Guidi, I.; Villa, C.; Bergamaschini, L.; Cortini, F.; Scalabrini, D.; Baron, P.; Vergani, C.; et al. Intrathecal Levels of IL-6, IL-11 and LIF in Alzheimer's Disease and Frontotemporal Lobar Degeneration. *J Neurol* **2008**, *255*, 539–544, doi:10.1007/s00415-008-0737-6.
46. Heese, K.; Nagai, Y.; Sawada, T. Induction of Rat L-Phosphoserine Phosphatase by Amyloid-Beta (1-42) Is Inhibited by Interleukin-11. *Neurosci Lett* **2000**, *288*, 37–40, doi:10.1016/s0304-3940(00)01197-6.
47. Sun, Y.; Song, X.; Geng, Z.; Xu, Y.; Xiao, L.; Chen, Y.; Li, B.; Shi, J.; Wang, L.; Wang, Y.; et al. IL-11 Ameliorates Oxidative Stress Damage in Neurons after Spinal Cord Injury by Activating the JAK/STAT Signaling Pathway. *International Immunopharmacology* **2024**, *127*, 111367, doi:10.1016/j.intimp.2023.111367.
48. Mehler, M.F.; Rozental, R.; Dougherty, M.; Spray, D.C.; Kessler, J.A. Cytokine Regulation of Neuronal Differentiation of Hippocampal Progenitor Cells. *Nature* **1993**, *362*, 62–65, doi:10.1038/362062a0.
49. Zhang, B.; Zhang, H.-X.; Shi, S.-T.; Bai, Y.-L.; Zhe, X.; Zhang, S.-J.; Li, Y.-J. Interleukin-11 Treatment Protected against Cerebral Ischemia/Reperfusion Injury. *Biomedicine & Pharmacotherapy* **2019**, *115*, 108816, doi:10.1016/j.biopha.2019.108816.
50. Spangenberg, E.; Severson, P.L.; Hohsfield, L.A.; Crapser, J.; Zhang, J.; Burton, E.A.; Zhang, Y.; Spevak, W.; Lin, J.; Phan, N.Y.; et al. Sustained Microglial Depletion with CSF1R Inhibitor Impairs Parenchymal Plaque Development in an Alzheimer's Disease Model. *Nat Commun* **2019**, *10*, 3758, doi:10.1038/s41467-019-11674-z.
51. Smith, A.M.; Gibbons, H.M.; Oldfield, R.L.; Bergin, P.M.; Mee, E.W.; Curtis, M.A.; Faull, R.L.M.; Dragunow, M. M-CSF Increases Proliferation and Phagocytosis While Modulating Receptor and Transcription Factor Expression in Adult Human Microglia. *J Neuroinflammation* **2013**, *10*, 85, doi:10.1186/1742-2094-10-85.
52. Li, C.; Chen, Y.; Luo, S.; Yang, Y.; Liu, X.; Li, S.; Ge, W.; Han, C. Differential Roles of Astrocytic CSF1 in Alzheimer's Disease and Cerebral Amyloid Angiopathy: Insights from Transcriptomic Analysis. *Aging Dis* **2024**, *16*, 3137–3153, doi:10.14336/AD.2024.10530.
53. Akiyama, H.; Nishimura, T.; Kondo, H.; Ikeda, K.; Hayashi, Y.; McGeer, P.L. Expression of the Receptor for Macrophage Colony Stimulating Factor by Brain Microglia and Its Upregulation in Brains of Patients with Alzheimer's Disease and Amyotrophic Lateral Sclerosis. *Brain Res* **1994**, *639*, 171–174, doi:10.1016/0006-8993(94)91779-5.

54. Walker, D.G.; Tang, T.M.; Lue, L.-F. Studies on Colony Stimulating Factor Receptor-1 and Ligands Colony Stimulating Factor-1 and Interleukin-34 in Alzheimer's Disease Brains and Human Microglia. *Front. Aging Neurosci.* **2017**, *9*, doi:10.3389/fnagi.2017.00244.
55. Boissonneault, V.; Filali, M.; Lessard, M.; Relton, J.; Wong, G.; Rivest, S. Powerful Beneficial Effects of Macrophage Colony-Stimulating Factor on β-Amyloid Deposition and Cognitive Impairment in Alzheimer's Disease. *Brain* **2009**, *132*, 1078–1092, doi:10.1093/brain/awn331.
56. Liu, Y.; Given, K.S.; Dickson, E.L.; Owens, G.P.; Macklin, W.B.; Bennett, J.L. Concentration-Dependent Effects of CSF1R Inhibitors on Oligodendrocyte Progenitor Cells Ex Vivo and in Vivo. *Exp Neurol* **2019**, *318*, 32–41, doi:10.1016/j.expneurol.2019.04.011.
57. Ou, W.; Yang, J.; Simanauskaite, J.; Choi, M.; Castellanos, D.M.; Chang, R.; Sun, J.; Jagadeesan, N.; Parfitt, K.D.; Cribbs, D.H.; et al. Biologic TNF-α Inhibitors Reduce Microgliosis, Neuronal Loss, and Tau Phosphorylation in a Transgenic Mouse Model of Tauopathy. *Journal of Neuroinflammation* **2021**, *18*, 312, doi:10.1186/s12974-021-02332-7.
58. Chang, R.; Knox, J.; Chang, J.; Derbedrossian, A.; Vasilevko, V.; Cribbs, D.; Boado, R.J.; Pardridge, W.M.; Sumbria, R.K. Blood-Brain Barrier Penetrating Biologic TNF-α Inhibitor for Alzheimer's Disease. *Mol Pharm* **2017**, *14*, 2340–2349, doi:10.1021/acs.molpharmaceut.7b00200.
59. Babić Leko, M.; Nikolac Perković, M.; Klepac, N.; Štrac, D.Š.; Borovečki, F.; Pivac, N.; Hof, P.R.; Šimić, G. IL-1β, IL-6, IL-10, and TNFα Single Nucleotide Polymorphisms in Human Influence the Susceptibility to Alzheimer's Disease Pathology. *J Alzheimers Dis* **2020**, *75*, 1029–1047, doi:10.3233/JAD-200056.
60. Khemka, V.K.; Ganguly, A.; Bagchi, D.; Ghosh, A.; Bir, A.; Biswas, A.; Chattopadhyay, S.; Chakrabarti, S. Raised Serum Proinflammatory Cytokines in Alzheimer's Disease with Depression. *Aging Dis* **2014**, *5*, 170–176, doi:10.14336/AD.2014.0500170.
61. Alvarez, X.A.; Franco, A.; Fernández-Novoa, L.; Cacabelos, R. Blood Levels of Histamine, IL-1 Beta, and TNF-Alpha in Patients with Mild to Moderate Alzheimer Disease. *Mol Chem Neuropathol* **1996**, *29*, 237–252, doi:10.1007/BF02815005.
62. Holmes, C.; Cunningham, C.; Zotova, E.; Woolford, J.; Dean, C.; Kerr, S.; Culliford, D.; Perry, V.H. Systemic Inflammation and Disease Progression in Alzheimer Disease. *Neurology* **2009**, *73*, 768–774, doi:10.1212/WNL.0b013e3181b6bb95.
63. Tarkowski, E.; Andreasen, N.; Tarkowski, A.; Blennow, K. Intrathecal Inflammation Precedes Development of Alzheimer's Disease. *J Neurol Neurosurg Psychiatry* **2003**, *74*, 1200–1205, doi:10.1136/jnnp.74.9.1200.
64. Bonotis, K.; Krikki, E.; Holeva, V.; Aggouridaki, C.; Costa, V.; Baloyannis, S. Systemic Immune Aberrations in Alzheimer's Disease Patients. *J Neuroimmunol* **2008**, *193*, 183–187, doi:10.1016/j.jneuroim.2007.10.020.
65. Lanzrein, A.S.; Johnston, C.M.; Perry, V.H.; Jobst, K.A.; King, E.M.; Smith, A.D. Longitudinal Study of Inflammatory Factors in Serum, Cerebrospinal Fluid, and Brain Tissue in Alzheimer Disease: Interleukin-1beta, Interleukin-6, Interleukin-1 Receptor Antagonist, Tumor Necrosis Factor-Alpha, the Soluble Tumor Necrosis Factor Receptors I and II, and Alpha1-Antichymotrypsin. *Alzheimer Dis Assoc Disord* **1998**, *12*, 215–227, doi:10.1097/00002093-199809000-00016.
66. De Luigi, A.; Fragiacomo, C.; Lucca, U.; Quadri, P.; Tettamanti, M.; Grazia De Simoni, M. Inflammatory Markers in Alzheimer's Disease and Multi-Infarct Dementia. *Mech Ageing Dev* **2001**, *122*, 1985–1995, doi:10.1016/s0047-6374(01)00313-x.
67. Kassner, S.S.; Bonaterra, G.A.; Kaiser, E.; Hildebrandt, W.; Metz, J.; Schröder, J.; Kinscherf, R. Novel Systemic Markers for Patients with Alzheimer Disease? - A Pilot Study. *Curr Alzheimer Res* **2008**, *5*, 358–366, doi:10.2174/156720508785132253.
68. Alvarez, A.; Cacabelos, R.; Sanpedro, C.; García-Fantini, M.; Aleixandre, M. Serum TNF-Alpha Levels Are Increased and Correlate Negatively with Free IGF-I in Alzheimer Disease. *Neurobiol Aging* **2007**, *28*, 533–536, doi:10.1016/j.neurobiolaging.2006.02.012.
69. Solerte, S.B.; Cravello, L.; Ferrari, E.; Fioravanti, M. Overproduction of IFN-Gamma and TNF-Alpha from Natural Killer (NK) Cells Is Associated with Abnormal NK Reactivity and Cognitive Derangement in Alzheimer's Disease. *Ann N Y Acad Sci* **2000**, *917*, 331–340, doi:10.1111/j.1749-6632.2000.tb05399.x.
70. Xu, C.; Wu, J.; Wu, Y.; Ren, Z.; Yao, Y.; Chen, G.; Fang, E.F.; Noh, J.H.; Liu, Y.U.; Wei, L.; et al. TNF-α-Dependent Neuronal Necroptosis Regulated in Alzheimer's Disease by Coordination of RIPK1-P62 Complex with Autophagic UVRAG. *Theranostics* **2021**, *11*, 9452–9469, doi:10.7150/thno.62376.
71. Serafini, S.; Ferretti, G.; Monterosso, P.; Angiolillo, A.; Di Costanzo, A.; Matrone, C. TNF-α Levels Are Increased in Patients with Subjective Cognitive Impairment and Are Negatively Correlated with β Amyloid-42. *Antioxidants*

2024, *13*, 216, doi:10.3390/antiox13020216.
72. Paouri, E.; Tzara, O.; Kartalou, G.-I.; Zenelak, S.; Georgopoulos, S. Peripheral Tumor Necrosis Factor-Alpha (TNF-α) Modulates Amyloid Pathology by Regulating Blood-Derived Immune Cells and Glial Response in the Brain of AD/TNF Transgenic Mice. *J Neurosci* **2017**, *37*, 5155–5171, doi:10.1523/JNEUROSCI.2484-16.2017.
73. Chakrabarti, S.; Prorok, T.; Roy, A.; Patel, D.; Dasarathi, S.; Pahan, K. Upregulation of IL-1 Receptor Antagonist by Aspirin in Glial Cells via Peroxisome Proliferator-Activated Receptor-Alpha1. *J Alzheimers Dis Rep* **2021**, *5*, 647–661, doi:10.3233/ADR-210026.
74. Barrientos, R.M.; Hein, A.M.; Frank, M.G.; Watkins, L.R.; Maier, S.F. Intracisternal Interleukin-1 Receptor Antagonist Prevents Postoperative Cognitive Decline and Neuroinflammatory Response in Aged Rats. *J. Neurosci.* **2012**, *32*, 14641–14648, doi:10.1523/JNEUROSCI.2173-12.2012.
75. Terrando, N.; Rei Fidalgo, A.; Vizcaychipi, M.; Cibelli, M.; Ma, D.; Monaco, C.; Feldmann, M.; Maze, M. The Impact of IL-1 Modulation on the Development of Lipopolysaccharide-Induced Cognitive Dysfunction. *Crit Care* **2010**, *14*, R88, doi:10.1186/cc9019.
76. Palin, K.; Bluthé, R.-M.; Verrier, D.; Tridon, V.; Dantzer, R.; Lestage, J. Interleukin-1β Mediates the Memory Impairment Associated with a Delayed Type Hypersensitivity Response to Bacillus Calmette–Guérin in the Rat Hippocampus. *Brain, Behavior, and Immunity* **2004**, *18*, 223–230, doi:10.1016/j.bbi.2003.09.002.
77. Faura, J.; Bustamante, A.; Penalba, A.; Giralt, D.; Simats, A.; Martínez-Sáez, E.; Alcolea, D.; Fortea, J.; Lleó, A.; Teunissen, C.E.; et al. CCL23: A Chemokine Associated with Progression from Mild Cognitive Impairment to Alzheimer's Disease. *J Alzheimers Dis* **2020**, *73*, 1585–1595, doi:10.3233/JAD-190753.
78. Shue, F.; White, L.J.; Hendrix, R.; Ulrich, J.; Henson, R.L.; Knight, W.; Martens, Y.A.; Wang, N.; Roy, B.; Starling, S.C.; et al. CSF Biomarkers of Immune Activation and Alzheimer's Disease for Predicting Cognitive Impairment Risk in the Elderly. *Science Advances* **2024**, *10*, eadk3674, doi:10.1126/sciadv.adk3674.
79. Laske, C.; Stellos, K.; Stransky, E.; Leyhe, T.; Gawaz, M. Decreased Plasma Levels of Granulocyte-Colony Stimulating Factor (G-CSF) in Patients with Early Alzheimer's Disease. *J Alzheimers Dis* **2009**, *17*, 115–123, doi:10.3233/JAD-2009-1017.
80. Sanchez-Ramos, J.; Song, S.; Sava, V.; Catlow, B.; Lin, X.; Mori, T.; Cao, C.; Arendash, G.W. Granulocyte Colony Stimulating Factor (G-CSF) Decreases Brain Amyloid Burden and Reverses Cognitive Impairment in Alzheimer's Mice. *Neuroscience* **2009**, *163*, 55–72, doi:10.1016/j.neuroscience.2009.05.071.
81. Prakash, A.; Medhi, B.; Chopra, K. Granulocyte Colony Stimulating Factor (GCSF) Improves Memory and Neurobehavior in an Amyloid-β Induced Experimental Model of Alzheimer's Disease. *Pharmacology Biochemistry and Behavior* **2013**, *110*, 46–57, doi:10.1016/j.pbb.2013.05.015.
82. Seripa, D.; Matera, M.G.; Forno, G.D.; Gravina, C.; Masullo, C.; Daniele, A.; Binetti, G.; Bonvicini, C.; Squitti, R.; Palermo, M.T.; et al. Genotypes and Haplotypes in the IL-1 Gene Cluster: Analysis of Two Genetically and Diagnostically Distinct Groups of Alzheimer Patients. *Neurobiology of Aging* **2005**, *26*, 455–464, doi:10.1016/j.neurobiolaging.2004.04.001.
83. Huang, C.-W.; Hsu, S.-W.; Tsai, S.-J.; Chen, N.-C.; Liu, M.-E.; Lee, C.-C.; Huang, S.-H.; Chang, W.-N.; Chang, Y.-T.; Tsai, W.-C.; et al. Genetic Effect of Interleukin-1 Beta (C-511T) Polymorphism on the Structural Covariance Network and White Matter Integrity in Alzheimer's Disease. *Journal of Neuroinflammation* **2017**, *14*, 12, doi:10.1186/s12974-017-0791-z.
84. Fidalgo, A.R.; Cibelli, M.; White, J.P.M.; Nagy, I.; Maze, M.; Ma, D. Systemic Inflammation Enhances Surgery-Induced Cognitive Dysfunction in Mice. *Neurosci Lett* **2011**, *498*, 63–66, doi:10.1016/j.neulet.2011.04.063.
85. Lopez-Rodriguez, A.B.; Hennessy, E.; Murray, C.L.; Nazmi, A.; Delaney, H.J.; Healy, D.; Fagan, S.G.; Rooney, M.; Stewart, E.; Lewis, A.; et al. Acute Systemic Inflammation Exacerbates Neuroinflammation in Alzheimer's Disease: IL-1β Drives Amplified Responses in Primed Astrocytes and Neuronal Network Dysfunction. *Alzheimers Dement* **2021**, *17*, 1735–1755, doi:10.1002/alz.12341.
86. Rivera-Escalera, F.; Pinney, J.J.; Owlett, L.; Ahmed, H.; Thakar, J.; Olschowka, J.A.; Elliott, M.R.; O'Banion, M.K. IL-1β-Driven Amyloid Plaque Clearance Is Associated with an Expansion of Transcriptionally Reprogrammed Microglia. *Journal of Neuroinflammation* **2019**, *16*, 261, doi:10.1186/s12974-019-1645-7.
87. Sheng, J.G.; Ito, K.; Skinner, R.D.; Mrak, R.E.; Rovnaghi, C.R.; Van Eldik, L.J.; Griffin, W.S. In Vivo and in Vitro Evidence Supporting a Role for the Inflammatory Cytokine Interleukin-1 as a Driving Force in Alzheimer Pathogenesis. *Neurobiol Aging* **1996**, *17*, 761–766, doi:10.1016/0197-4580(96)00104-2.
88. Balducci, C.; Frasca, A.; Zotti, M.; La Vitola, P.; Mhillaj, E.; Grigoli, E.; Iacobellis, M.; Grandi, F.; Messa, M.; Colombo, L.; et al. Toll-like Receptor 4-Dependent Glial Cell Activation Mediates the Impairment in Memory Establishment Induced by β-Amyloid Oligomers in an Acute Mouse Model of Alzheimer's Disease. *Brain Behav*

Immun **2017**, *60*, 188–197, doi:10.1016/j.bbi.2016.10.012.
89. Kitazawa, M.; Cheng, D.; Tsukamoto, M.R.; Koike, M.A.; Wes, P.D.; Vasilevko, V.; Cribbs, D.H.; LaFerla, F.M. Blocking IL-1 Signaling Rescues Cognition, Attenuates Tau Pathology, and Restores Neuronal β-Catenin Pathway Function in an Alzheimer's Disease Model. *J Immunol* **2011**, *187*, 6539–6549, doi:10.4049/jimmunol.1100620.
90. Ghosh, S.; Wu, M.D.; Shaftel, S.S.; Kyrkanides, S.; LaFerla, F.M.; Olschowka, J.A.; O'Banion, M.K. Sustained Interleukin-1β Overexpression Exacerbates Tau Pathology Despite Reduced Amyloid Burden in an Alzheimer's Mouse Model. *J. Neurosci.* **2013**, *33*, 5053–5064.
91. Viviani, B.; Bartesaghi, S.; Gardoni, F.; Vezzani, A.; Behrens, M.M.; Bartfai, T.; Binaglia, M.; Corsini, E.; Di Luca, M.; Galli, C.L.; et al. Interleukin-1beta Enhances NMDA Receptor-Mediated Intracellular Calcium Increase through Activation of the Src Family of Kinases. *J Neurosci* **2003**, *23*, 8692–8700, doi:10.1523/JNEUROSCI.23-25-08692.2003.
92. Benzing, W.C.; Wujek, J.R.; Ward, E.K.; Shaffer, D.; Ashe, K.H.; Younkin, S.G.; Brunden, K.R. Evidence for Glial-Mediated Inflammation in Aged APP(SW) Transgenic Mice. *Neurobiol Aging* **1999**, *20*, 581–589, doi:10.1016/s0197-4580(99)00065-2.
93. Nie, J.; Fang, Y.; Chen, Y.; Aidina, A.; Qiu, Q.; Zhao, L.; Liu, X.; Sun, L.; Li, Y.; Zhong, C.; et al. Characteristics of Dysregulated Proinflammatory Cytokines and Cognitive Dysfunction in Late-Life Depression and Amnestic Mild Cognitive Impairment. *Front Immunol* **2021**, *12*, 803633, doi:10.3389/fimmu.2021.803633.
94. Doroszkiewicz, J.; Kulczyńska-Przybik, A.; Dulewicz, M.; Mroczko, J.; Borawska, R.; Słowik, A.; Zetterberg, H.; Hanrieder, J.; Blennow, K.; Mroczko, B. Associations between Microglia and Astrocytic Proteins and Tau Biomarkers across the Continuum of Alzheimer's Disease. *Int J Mol Sci* **2024**, *25*, 7543, doi:10.3390/ijms25147543.
